# Inter-element variation in the bone histology of *Anteosaurus* (Dinocephalia, Anteosauridae) from the *Tapinocephalus* Assemblage Zone of the Karoo Basin of South Africa

**DOI:** 10.7717/peerj.12082

**Published:** 2021-09-08

**Authors:** Mohd Shafi Bhat, Christen D. Shelton, Anusuya Chinsamy

**Affiliations:** 1 Department of Biological Sciences, University of Cape Town, Cape Town, South Africa; 2 Natural History Department, New Jersey State Museum, Trenton, New Jersey, United States; 3 Biology/Mathematics & Physical Science Departments, Rogers State University, Claremore, Oklahoma, USA

**Keywords:** Abrahamskraal formation, Beaufort group, Bone microstructure, Middle Permian, Dinocephalia, Synapsida

## Abstract

Despite its abundance in the Permian fossil record of South Africa, little is known about the life history of *Anteosaurus*. Here we examine the bone microstructure of multiple skeletal elements of *Anteosaurus* from the *Tapinocephalus* Assemblage Zone of the Karoo Basin. The bone histology of *Anteosaurus magnificus* reveals that the cortex is composed of highly vascularized, uninterrupted fibrolamellar bone tissue surrounding the inner spongy medullary region. However, the histology of two ribs and a previously described femur of another *Anteosaurus* taxon revealed an interrupted growth pattern with lines of arrested growth and peripheral rest lines occurring in the compacta, indicating periodic pauses in growth possibly linked to the slowing down of growth during maturity. Given that the fibula of the same individual has well-vascularised fibrolamellar bone tissue without any growth marks in the cortex; this suggests variation in skeletal growth. Based on our histological results, three growth dynamic stages are deduced for the genus *Anteosaurus*: (i) the earliest growth stage is represented by the predominance of highly vascularized, uninterrupted fibrolamellar bone tissue in the inner cortex, which suggests rapid periosteal bone deposition during early ontogeny; (ii) the next stage of growth shows periodic interruptions in the bone deposition as indicated by the deposition of lines of arrested growth; (iii) the third stage shows the development of lamellar bone tissue with rest lines in the peripheral part of the cortex suggesting a slowing down of growth prior to death. Most of the skeletal elements are characterized by thick bone walls, extensive secondary reconstruction and the complete infilling of the medullary cavity. However, the radius and a previously studied femur have open medullary cavities with struts of bony trabeculae. Based on histologic structures and comparisons with extant taxa, it is likely that *Anteosaurus* may have been more terrestrial as its osteology point towards terrestriality, but it may have occasionally inhabited ephemeral pools like modern semi-aquatic *Hippopotamus*.

## Introduction

The anteosaurs, a monophyletic group of dinocephalians (Therapsida: Dinocephalia: Anteosauria), first appeared during the Middle Permian (Guadalupian) and formed a key component of the terrestrial tetrapod fauna (Rubidge, 1995; Rubidge & Sidor, 2001; Kammerer, 2011; Cisneros et al., 2012; Kemp, 1982, 2012; Smith, Rubidge & Walt, 2012). The family Anteosauridae comprises two major clades, Syodontinae (*Australosyodon*, *Notosyodon* and *Syodon*) and Anteosaurinae (*Anteosaurus*, *Sinophoneus* and *Titanophoneus*) with Russian taxa *Archaeosyodon* and *Microsyodon* representing the most basal anteosaurs (Kammerer, 2011). Liu (2013) revised the phylogeny of Anteosauridae using the modified character lists and data matrices of Kammerer (2011) and Cisneros et al. (2012) and recovered *Sinophoneus* as a basal anteosaur, falling outside the clade Anteosaurinae. Anteosaurs are known from Middle Permian rocks of both Laurasia (Efremov, 1954; Tchudinov, 1968; Ivachnenko, 1995; Li, Rubidge & Cheng, 1996; Cheng & Ji, 1996; Cheng & Li, 1997; Golubev, 2015) and Gondwana (Owen & Bain, 1845; Owen, 1879; Watson, 1921; Broom, 1929; Boonstra, 1954, 1969; Rubidge, 1994; Cisneros et al., 2012; Kruger, Rubidge & Abdala, 2018). There are more than eight valid genera of anteosaurs (King, 1988; Kammerer, 2011; Kemp, 2012; Cisneros et al., 2012; Liu, 2013) but only two, *Australosyodon* and *Anteosaurus*, are represented in the Beaufort Group of the Karoo Supergroup of South Africa (Boonstra, 1969; King, 1988; Rubidge, 1994; Kemp, 2012; Kruger, Rubidge & Abdala, 2018). They were the largest apex predators during the Middle Permian Period; however, by the end of the *Tapinocephalus* Assemblage Zone (Smith & Keyser, 1995; Kemp, 2012; Day & Rubidge, 2020; Day & Smith, 2020), they along with other dinocephalians completely disappear from the fossil record leaving no descendants (Boonstra, 1971; Kemp, 1982, 2005, 2012). Their extinction within the Karoo Basin is linked either to the increasing aridification or to the loss of their food sources (Bond et al., 2010; Rey et al., 2018; Day & Rubidge, 2021). Anteosaurs are characterized by large pachyostotic skulls, which range in length from 280 to 805 mm (Boonstra, 1954; Kemp, 1982; Kammerer, 2011; Kruger, Rubidge & Abdala, 2018). The members of the family Anteosauridae are diagnosed by combined synapomorphic features: anterodorsally canted premaxilla, convex ventral maxillary margin, ‘scroll’ vomers, quadrate rami of the pterygoid that bifurcate the anterior margin of the basisphenoid, a ridge on the jugal-lacrimal suture, and a ‘scoop’-shaped (strongly anteroventrally curved) postorbital bar (Kemp, 1982; Ivakhnenko, 2008; Kammerer, 2011).

Anteosaurs were fully terrestrial animals feeding on large tapinocephalid dinocephalians, bull-sized armored pareiasaurs and even scavenging on kills made by the lycosuchid and scylacosaurid therocephalians (Boonstra, 1955; Rubidge, 1995; Lee, 1997; Nicolas & Rubidge, 2010; Kammerer, 2011; Kemp, 2012; Canoville, Thomas & Chinsamy, 2014). They have also been considered riparian, obligate fish-eaters (Boonstra, 1955, 1962; Ivakhnenko, 2001, 2003, 2008), or even amphibious (Olson, 1962; King, 1988; Ivakhnenko, 2008). Kammerer (2011) supported a fully terrestrial lifestyle for anteosaurs as they possessed the heaviest skulls of all the carnivorous synapsids, and were equipped with large canines and incisors, that would have been effective for preying on large land animals (Sennikov, 1996; Van Valkenburgh & Jenkins, 2002). Canoville, Thomas & Chinsamy (2014) performed isotopic analysis of the teeth of dinocephalians and pointed out that most of the dinocephalians had lower oxygen isotope compositions than contemporaneous pareiasaurs and therocephalians. They further suggested that dinocephalians and pareiasaurs inhabited different ecological niches and that the herbivorous pareiasaurs may have shared a terrestrial habitat with carnivorous therocephalians. However, their isotopic differences also reflected higher water turnover rates for dinocephalians suggesting niche partitioning (Canoville, Thomas & Chinsamy, 2014). Later, Rey et al. (2020) supported a terrestrial adaptation for *Anteosaurus* even though their oxygen values point towards water dependency. Since their sample size was small, they were unable to confirm whether anteosaurs were riparian or more in-land dwellers. Recently Benoit et al. (2021) concluded that anteosaurs were agile predators, based on the enlarged fossa for the floccular lobe of the cerebellum and semicircular canals of the inner ear.

Bone microanatomical studies of tetrapods have demonstrated that bone architecture (microanatomy) and internal tissue structure (histology) varies according to lifestyle adaptations (Wall, 1983; Chinsamy, 1991; Ray, Chinsamy & Bandyopadhyay, 2005; Gray et al., 2007; Hayashi et al., 2013; Houssaye et al., 2016; Canoville, de Buffrénil & Laurin, 2016; Canoville & Chinsamy, 2017). Furthermore, bone histology of extant and extinct vertebrates provides direct assessment of life history and the biology of an animal (de Ricqlès, 1969, 1972; Francillon-Vieillot et al., 1990; Chinsamy, 1997; Horner, de Ricqlès & Padian, 1999, 2000; Chinsamy-Turan, 2005, 2012; Klein & Sander, 2008; Chinsamy et al., 2013, 2019, 2020; Woodward, Horner & Farlow, 2014; Woodward et al., 2015; Woodward et al., 2020; Huttenlocker & Botha-Brink, 2014; Botha-Brink, Soares & Martinelli, 2018; Cullen et al., 2020; Huttenlocker & Shelton, 2020; Botha, 2020). Several studies have examined the bone histology of the non-mammalian therapsids (see Chinsamy-Turan, 2012 and references therein), however, except for the early work of de Ricqlès (1972), dinocephalians have been relatively under-studied. Recent histology research on *Titanosuchus* led to the identification of osteomyelitis in a femur of *Jonkeria parva* (Shelton, Chinsamy & Rothschild, 2019). More recently, additional bone histology studies have been conducted on *Jonkeria* (Bhat, Shelton & Chinsamy, in press (a)) and have shown that these fast growing omnivorous animals were adapted for a semi-aquatic lifestyle, like modern graviportal *Hippopotamus*. Subsequently, a comprehensive histological study of various limb bones of several herbivorous dinocephalian taxa found richly vascularized fibrolamellar bone tissue, as well as extensively developed medullary spongiosa, and supported the semi-aquatic lifestyle hypothesis for herbivorous dinocephalian taxa Bhat, Shelton & Chinsamy, in press (b).

Since tetrapods display a wide range of histological characteristics (*e.g*., Horner, de Ricqlès & Padian, 1999, 2000; Ray & Chinsamy, 2004; Ray, Botha & Chinsamy, 2004; Chinsamy-Turan, 2005, 2012), as well as bone depositional rates (*e.g*., Amprino, 1947; de Margerie, Cubo & Castanet, 2002; de Margerie et al., 2004; Starck & Chinsamy, 2002), multi-element studies of individuals of a particular species provide a better assessment of their growth patterns, lifestyle habits and various aspects of their life history (Horner, de Ricqlès & Padian, 1999, 2000; Botha & Chinsamy, 2004, 2005; Chinsamy-Turan, 2005, 2012; Ray, Mukherjee & Bandyopadhyay, 2009; Woodward et al., 2015). This study represents the first histological and microanatomical study of multiple skeletal elements of two *Anteosaurus* taxa. Our study aims to assess the inter-elemental variation in their bone histology as well as to shed light on their lifestyle adaptations.

## Materials & Methods

**Figure 1:**
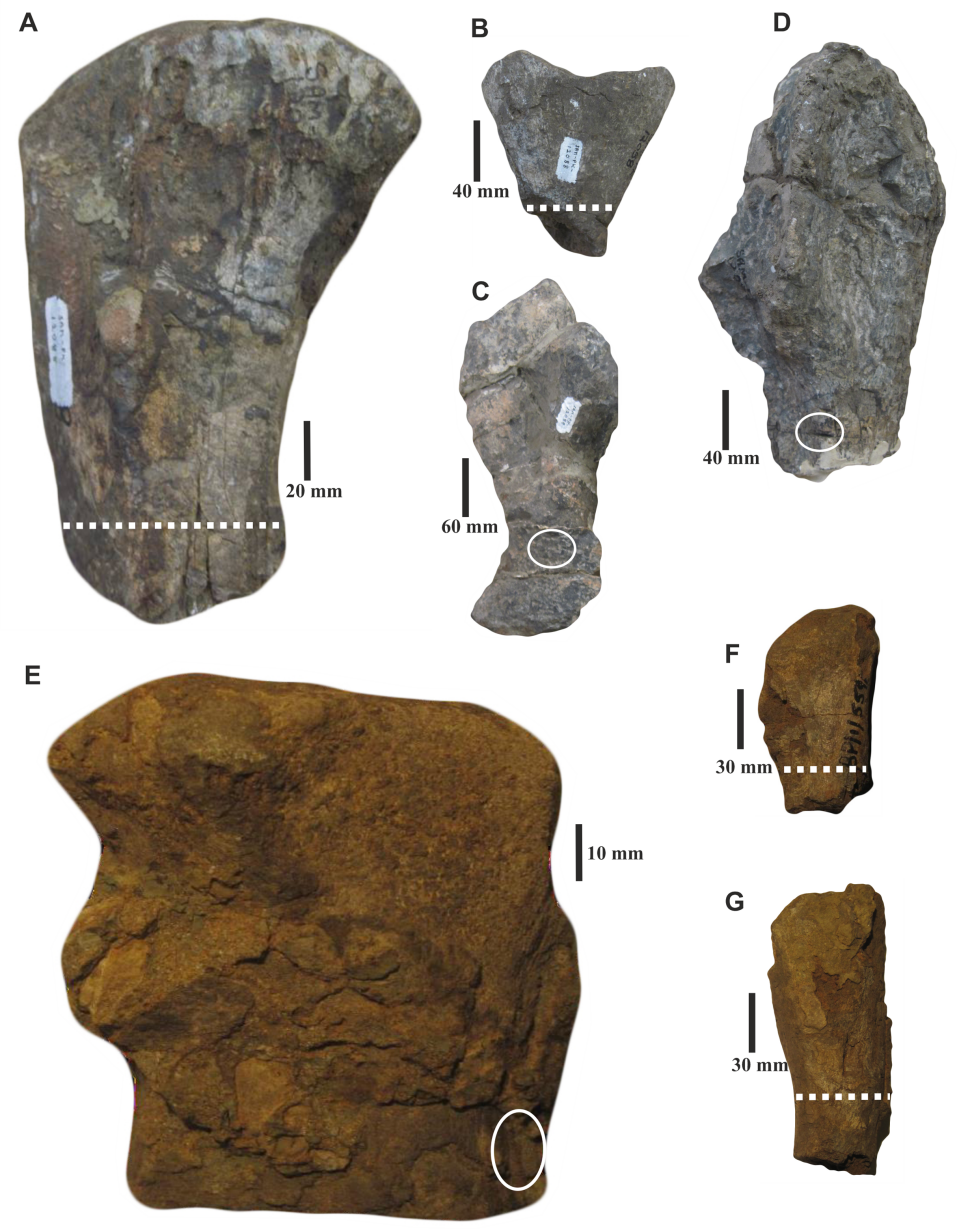
Skeletal elements of *Anteosaurus* specimens studied. (A) Proximal femur (SAM-PK-12088a). (B) Proximal radius (SAM-PK-12088b). (C) Ulna (SAM-PK-12088c). (D) Ulna head (SAM-PK-12088d). (E) Fibula fragment (BP/1/5591b). (F) Proximal rib (BP/1/5591c). (G) Rib (BP/1/5591d). The dotted lines and circles indicate the histological section planes and transverse cores.

The specimens studied here were excavated from the *Tapinocephalus* Assemblage Zone of the South African Karoo Basin (Boonstra, 1969). For our analysis, several skeletal elements (femora, radius, ulnae, fibula, and ribs; Fig. 1) were examined to assess inter-elemental histological variability. These skeletal elements are positively identified at species or generic levels (Table 1; references therein). In the text, different elements of the same individual have the same specimen numbers but are differentiated by the suffixes of a/b/c. The skeletal elements with specimen number SAM-PK-12088 belong to *Anteosaurus magnificus* whereas the fibula (BP/1/5591b) and ribs (BP/1/5591c–d) belong to another *Anteosaurus* taxon. All specimens were obtained from Iziko South African Museums, Cape Town, and Evolutionary Studies Institute (formerly the Bernard Price Institute) at the University of the Witwatersrand, Johannesburg, South Africa. Permission to section the fossils was obtained from the South African Heritage Resources Agency (SAHRA; Permits 2076, 2131, and 3752–4658).

**Table 1:** Skeletal elements of *Anteosaurus* specimens studied.

Specimen number		Skeletal element	Taxon	Locality	Section type
SAM-PK-12088	a	Femur	*Anteosaurus magnificus*	Sewefontein, Prince Albert, *Tapinocephalus* Assemblage Zone, Beaufort Group, Karoo Supergroup, South Africa.	Transverse
	b	Proximal radius			Transverse
	c	Ulna			Transverse core
	d	Ulna head			Transverse core
BP/1/5591	b	Fibula	*Anteosaurus* sp.	Rheboksfontein *Tapinocephalus* Assemblage Zone, Beaufort Group, Karoo Supergroup, South Africa.	Transverse core
	c	Proximal rib			Transverse
	d	Rib midshaft			

**Notes:**

All the material was recovered from the *Tapinocephalus* Assemblage Zone (Beaufort Group, Karoo Supergroup), South Africa.

Abbreviations: SAM, Iziko South African Museums Cape Town, South Africa; BP, Evolutionary Sciences Institute (previously Bernard Price Institute);University of the Witwatersrand, Johannesburg, South Africa. Source of information: Boonstra (1955, 1969); King (1988).

Transverse sections were prepared from midshaft levels wherever possible as these are the regions of the bone that undergo the least amount of secondary remodeling (Enlow, 1963; Chinsamy, 1995; Chinsamy-Turan, 2005) and limb bones were preferentially selected because they retain the best record of growth (Francillon-Vieillot et al., 1990; Chinsamy-Turan, 2005; Bhat, Chinsamy & Parkington, 2019). Both stylopodial (femur) and zeugopodial (radius, ulna; fibula) bones were sampled as they can show different ecological signals and respond differently to any change in the habitat (de Buffrénil & Schoevaert, 1989; de Margerie et al., 2005; Canoville & Laurin, 2010; Quemeneur, de Buffrénil & Laurin, 2013). We also sectioned non-weight bearing bones (ribs) to investigate whether they exhibit a better growth mark record in their proximal ends like those of sauropod dinosaurs (Stein & Sander, 2009; Waskow & Sander, 2014). The destructive nature of histological analyses and the scarcity of complete specimens prohibited the sectioning of a large number of bones; however, an optimal sample was obtained by selecting diagnostic though incomplete skeletal elements.

Thin sections of long bones were petrographically prepared using cutting and grinding techniques following Chinsamy & Raath (1992). Considering the technical challenge of sectioning large dinocephalian skeletal elements, limb bones were sampled by the hydraulic coring method using a drill with a one cm diamond encrusted coring bit or cut using a Dremel Precision Tool, following the standard procedures outlined in Stein & Sander (2009). Core drilling was preferentially performed in an area that would cause the least amount of damage to the anatomy of the element. Note that permission was granted to section skeletal elements only in clearly specified areas (*e.g.*, ends of the bone/broken regions) and therefore this prevented us from sectioning both proximal and midshaft regions from the same element. After the cores were obtained, the holes were infilled with plaster to preserve the overall morphology of the bone. Depending on the fossil itself, each core was either embedded in an epoxy resin (EpoxAcast 690 and/or Struers Epofix; Chinsamy & Raath, 1992; Chinsamy-Turan, 2005) or subjected to direct cutting/slicing along the preferred direction. The coring, sectioning, embedding and thin sectioning, as well as the microscopy were performed in the thin sectioning laboratory of the Palaeobiology Research Group at the Department of Biological Sciences, University of Cape Town. The embedded bones were mounted on frosted glass slides and thin sectioned using a Struers Accutom-50 and ground and polished using carborundum (silicon carbide) discs of various grit sizes (400–1,200 μm). This was followed by a final polish on a lap wheel with a velvet cloth using aluminium oxide (Al₂O₃) solution. The final thickness of the section was between 45–50 microns; this proved to be optimum for our analyses. Bones were processed in serial sections in case of slide breakages or loss of slide. All prepared sections were studied and photographed using a digital compact camera Nikon DS-Fi1 mounted on Carl Zeiss Axio Lab A1 polarizing microscope. Histological nomenclature follows that of Francillon-Vieillot et al. (1990) and Chinsamy-Turan (2005, 2012).

## Results

**Figure 2:**
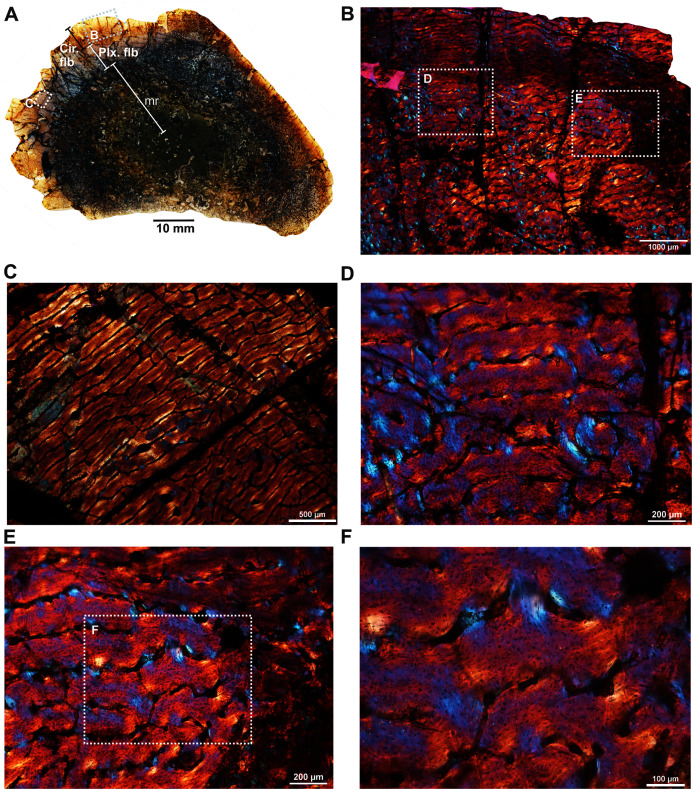
Transverse sections of the femur (SAM-PK-12088a) of *Anteosaurus magnificus*. Figs. 1B–1F were imaged under cross-polarized light with lambda compensator. (A) Transverse section showing the thick outer cortex and medullary spongiosa. (B) Magnified view of framed area in ‘A’ showing highly vascularized circumferential fibrolamellar bone. (C) Magnified view of framed area in ‘A’ showing plexiform fibrolamellar bone. (D) Magnified view of framed area in ‘B’ showing fibrolamellar bone tissue with woven matrix in the outer cortex. (E) Magnified view of framed area in ‘B’ showing the transition between inner plexiform and outer continuous circumferential vascular canals. (F) Magnified view of framed area in ‘E’ showing woven bone with dense lacunae. Abbreviations: cir. flb, circumferential fibrolamellar bone; plx. flb, plexiform fibrolamellar bone; mr, medullary region.

The femur (SAM-PK-12088a; Fig. 1A) belongs to *A*. *magnificus* (Table 1). The histology is well preserved even though the section is damaged by numerous cracks (Fig. 2A). The maximum diameter of the cross-section is approximately 73 mm. It has a distinct, outer cortex and medullary region partially infilled by trabeculae (Fig. 2A). The bone wall is thick, but the extensive resorption of vascular canals gives the inner cortex a spongy aspect. The overall cross-section is divided into three regions: inner spongy bone, a middle plexiform layer and an outer band of circumferential fibrolamellar bone. The medullary region is large, and the perimedullary region consists of unevenly distributed cancellous bone that grades into the more compacted mid-cortex (Fig. 2A). The predominant bone tissue of the outer cortex is highly vascularized uninterrupted fibrolamellar bone tissue (Figs. 2B–2F) with a woven matrix as defined by fiber orientation, globular and randomly distributed osteocyte lacunae (Figs. 2D–2F). Fibrolamellar bone deposition continues up to the peripheral margin of the bone without any decrease in vascularization or change in the tissue type (Fig. 2B). The primary osteons in the peripheral cortex have a circumferential orientation (Figs. 2B, 2D–2F). A change in vascularity to a more plexiform pattern with radial and circumferential channels is observed in the inner mid-cortex (Figs. 2A, 2C). Growth marks are not present in the compacta.

**Figure 3:**
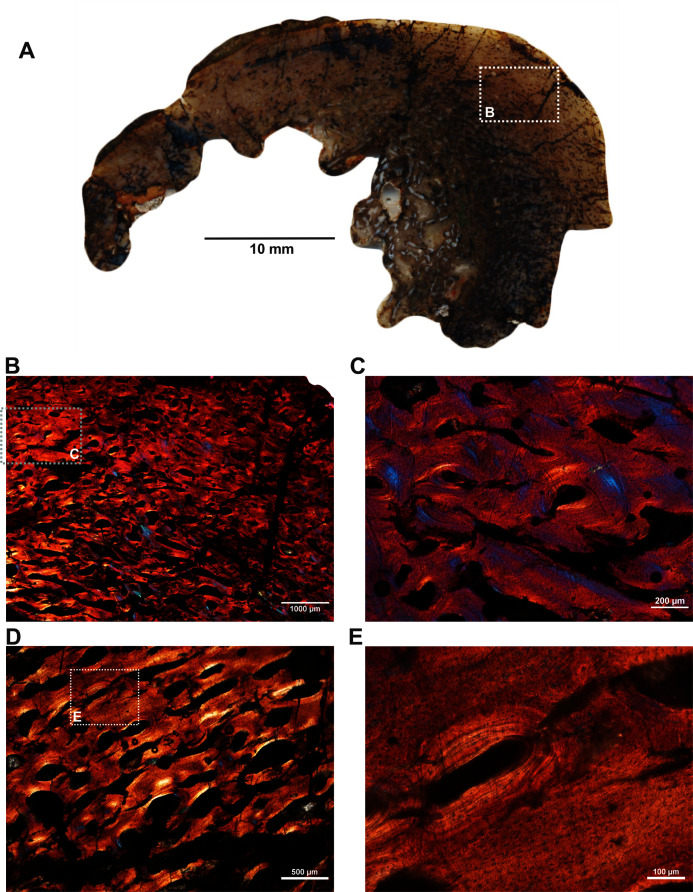
Transverse sections of the proximal radius (SAM-PK-12088b) of *Anteosaurus magnificus*. Figs. 2B–2E were imaged under cross-polarized light with lambda compensator. (A), transverse section of the radius under ordinary light showing a thick outer cortex and an open medullary cavity with thick struts of trabeculae. (B) Magnified view of framed area in ‘A’ showing highly porous outer cortex. (C) Magnified view of framed area in ‘B’ showing woven bone matrix and numerous primary osteons. (D) Large erosional spaces and centripetal bone deposition. (E) Magnified view of framed area in ‘D’ showing deposition of lamellar bone around the vascular canals.

A transverse section of the *A. magnificus* radius (SAM-PK-12088b; Fig. 1B) displays an open medullary cavity with thick struts of trabeculae (Fig. 3A). Given that the section is from the epiphyseal region, the medullary cavity is quite irregular and resorptive (Fig. 3A). Like the femur SAM-PK-12088a (Fig. 2A), the outer cortex is comprised of highly vascularized fibrolamellar bone tissue (Figs. 3C–3D) with a woven matrix as shown by the orientation of the collagen fibers and the profuse, globular, haphazardly oriented osteocyte lacunae. The vascular channels within the outer cortex are mostly longitudinally orientated and arranged circumferentially (Figs. 3B–3C), and they are surrounded by osteonal deposits forming primary osteons (Fig. 3C). The size and density of vascular canals are mostly consistent throughout the compacta (Fig. 3B), although, secondary reconstruction is extensive in the perimedullary region and has resulted in numerous enlarged cavities (Fig. 3D). Many of these erosional spaces are secondarily infilled by centripetally deposited lamellar bone (Fig. 3E).

**Figure 4:**
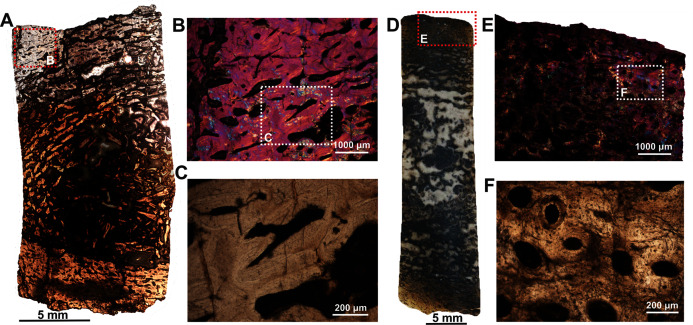
Histological thin section of a core of the ulnae (SAM-PK-12088c–d) of *Anteosaurus magnificus* under ordinary light (A, C, D, F) and under cross-polarized light with lambda compensator (B, E). (A) Overall view of the core showing highly vascularized outer cortex surrounding infilled medullary region. (B) Magnified view of framed area in ‘A’ showing circumferentially arranged vascular canals and large resorption cavities. (C) Magnified view of framed area in ‘B’ showing large erosional spaces encircled by lamellar bone tissue. (D) Overall view of the core showing highly vascularized outer cortex surrounding partially infilled medullary region. (E) Magnified view of framed area in ‘D’ showing circumferential arranged longitudinal vascular canals. (F) Magnified view of framed area in ‘E’ showing large vascular canals encircled by lamellar bone tissue.

Two ulnae (SAM-PK-12088c–d; Figs. 1C–1D) of *A. magnificus* were sectioned at the distal epiphyseal (Figs. 4A–4C) and midshaft regions (Figs. 4D–4F). They exhibited similar histological features as both are composed of highly vascularized cortex with an inner medullary region infilled by a dense network of bony trabeculae. Near the distal epiphyseal end (Fig. 4A), the bone is highly remodeled with many secondarily enlarged erosional cavities showing centripetal deposits of lamellar bone tissue (Figs. 4B–4C). The cross-section of the ulna at midshaft region (Fig. 4D) shows a compact bone with a large, partially filled medullary region. The medullary region has numerous islands of bony trabeculae. Circumferentially arranged longitudinal vascular canals are abundant in the compacta (Figs. 4E–4F).

**Figure 5:**
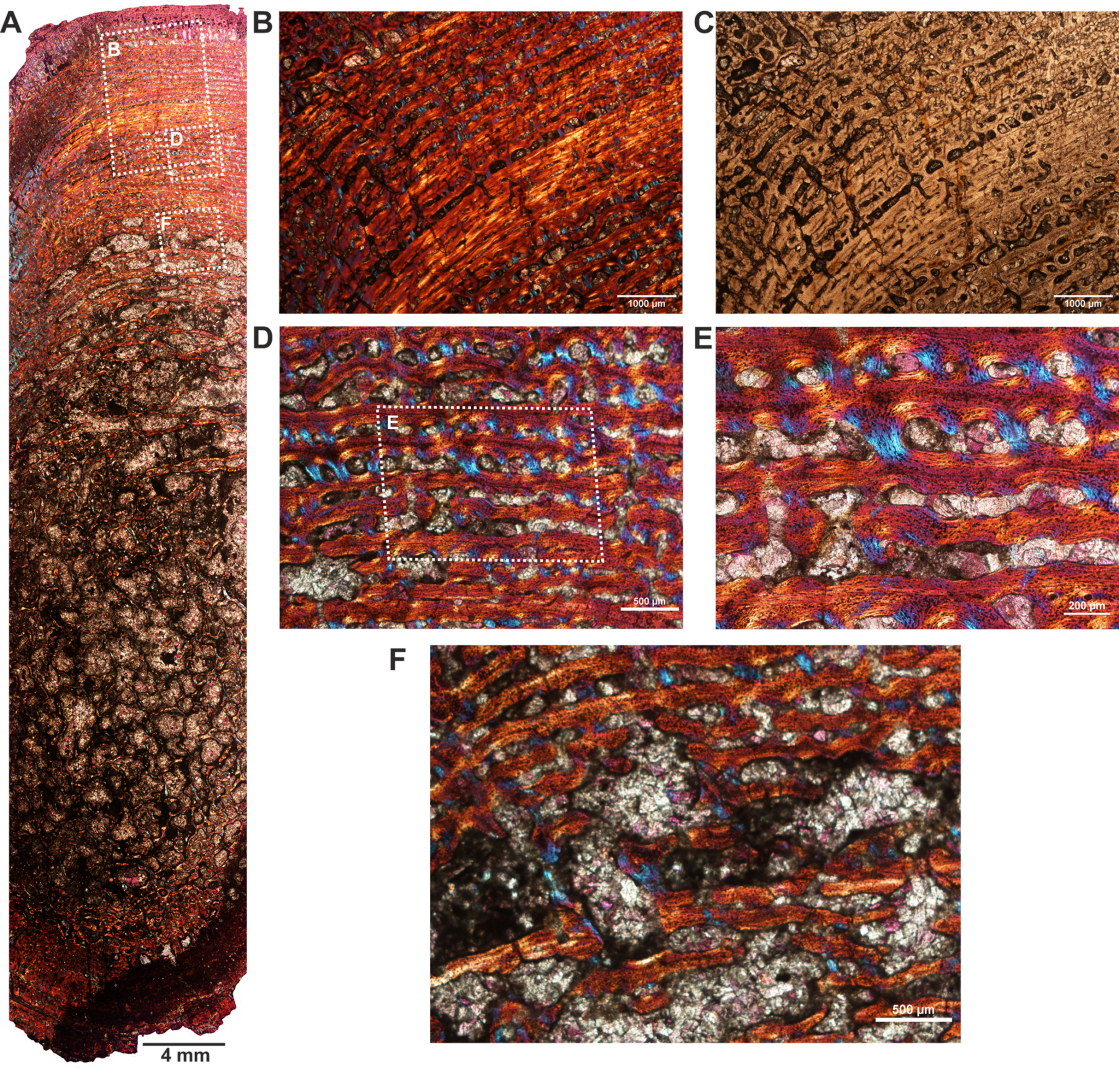
Histological thin section of a core of the fibula (BP/1/5591b) of *Anteosaurus* under cross-polarized light with lambda compensator (A, B, D, E, F), and under ordinary light (C). (A) Overall view of the core showing highly vascularized fibrolamellar bone tissue in the outer cortex surrounding infilled medullary cavity. Note: the trabecular density in the perimedullary region and mineral cement filling the large open spaces. (B) Magnified view of framed area in ‘A’ showing circumferentially oriented laminar vascular canals. (C) Same as previous image, but under ordinary light. (D) Magnified view of framed area in ‘A’ showing circumferential vascular canals and resorption between individual channels. (E) Magnified view of framed area in ‘D’ showing high density of osteocyte lacunae and large resorption between channels. (F) Magnified view of framed area in ‘A’ showing large resorption cavities with mineral cement.

The fibula (BP/1/5591b; Fig. 1E) of another *Anteosaurus* taxon shows an inner medullary region infilled by a dense network of bony trabeculae (Fig. 5A) while the pore spaces are occupied by diagenetic minerals (Fig. 5A, 5F). The outer margin of the medullary cavity and the inner medullary region have islands of trabeculae with a woven matrix (Fig. 5F). Resorption is intense in the inner cortex resulting in large erosional cavities (Fig. 5A). The size of the eroded cavities decreases from the inner cortex to the peripheral cortex (Fig. 5A). Overall, the compact bone tissue is laminar fibrolamellar with woven matrix (Figs. 5D–5E). The vascularization pattern is laminar towards the inner cortex whereas subperiosteally there tends to be more radial anastomoses between the circumferentially organized vascular canals (Figs. 5B–5C).

**Figure 6:**
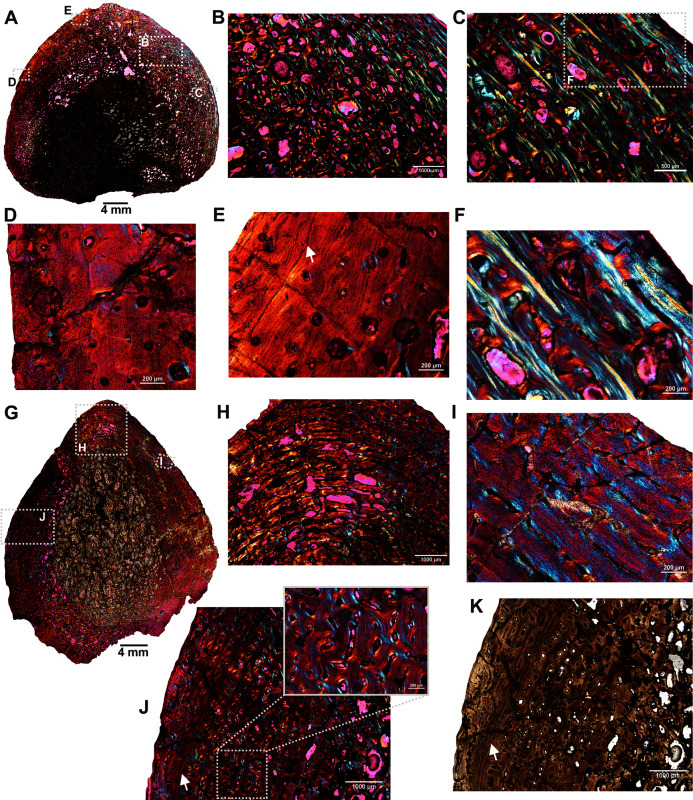
Transverse sections of the ribs (BP/1/5591c-d) of *Anteosaurus* under cross-polarized light with lambda compensator (A–J), and under ordinary light (K). (A) Diaphyseal cross-section at proximal end of the rib (BP/1/5591c) showing highly remodeled cortex surrounding cancellous medullary region. Note: the progressive transition from interior thin trabeculae to thick outer but porous cortex. (B) Magnified view of framed area in ‘A’ showing large erosional spaces. (C) Magnified view of framed area in ‘A’ showing lamellar bone tissue in between erosional spaces. (D) Magnified view of framed area in ‘A’ showing thin layer of fibrolamellar bone tissue towards the periphery (*i.e.*, left side of the image) and inner lamellar bone tissue (*i.e.*, right side of the image). (E) Magnified view of framed area in ‘A’ showing inner lamellar bone tissue with numerous rest lines. Note: a LAG (white arrow) towards peripheral cortex. (F) Highly vascularized fibrolamellar bone tissue with woven matrix in the outer cortex. Note: the density of osteocyte lacunae towards periphery and lamellar bone tissue between open spaces. (G) Diaphyseal cross-section at midshaft level (BP/1/5591d) showing highly vascular fibrolamellar outer cortex surrounding cancellous medullary region. Note: the variation of bone tissue and cortical porosity between sections of the two ribs of the same individual at different levels of the shaft. (H) Magnified view of framed area in ‘G’ showing large erosional spaces and circumferential laminar vascular canals. (I) Magnified view of framed area in ‘G’ showing fibrolamellar bone tissue with primary osteons. (J) Magnified view of framed area in ‘G’ showing highly vascular fibrolamellar bone tissue with woven matrix (inset). Note: a LAG (white arrow) towards peripheral cortex and the density of osteocyte lacunae towards periphery. (K) Same as previous image, but under ordinary light.

Two ribs (BP/1/5591c–d; Figs. 1F–1G) of *Anteosaurus* from the same individual were sectioned; one rib (BP/1/5591c; Fig. 1F) was sectioned at the proximal end (Figs. 6A–6F) and the other rib (BP/1/5591d; Fig. 1G) at the midshaft region (Figs. 6G–6K). The cortical thickness and overall shape of the section varies in different regions of the rib shaft (Figs. 6A, 6G). Near the proximal end (Fig. 6A), the bone is highly remodeled with many secondarily enlarged erosional cavities (Fig. 6B). Narrow bands of lamellar bone tissue occur between open spaces in the inner cortex (Figs. 6B–6C) whereas subperiosteally fibrolamellar bone tissue forms a thin peripheral layer (Figs. 6D, 6F). Several closely spaced rest lines and a few primary osteons are present in the subperiosteal region (Fig. 6E). The cross-section of rib BP/1/5591d taken at the midshaft level (Fig. 6G) displays a thick outer cortex of highly vascularized fibrolamellar bone tissue. However, like the proximal section, the inner cortex is spongy comprising of bony trabeculae (Fig. 6G). The outer cortex has numerous longitudinal primary osteons arranged circumferentially within the fibrolamellar bone preserving dense randomly organized globular osteocyte lacunae (Figs. 6H–6K). Circumferentially oriented longitudinal canals are dominant in the compacta (Figs. 6H–6K). A few localized radial and reticular organized vascular canals are visible (Figs. 6H, 6K). A line of arrested growth (LAG) is present in the outer peripheral cortex (Figs. 6J–6K).

## Discussion

### Growth dynamics

Except for the proximal rib BP/1/5591c, all the bones exhibited fibrolamellar tissue to varying degrees in the inner cortices, suggesting that the early stage of growth was fast and resulted in the deposition of a more rapidly formed bone tissue (Amprino, 1947; Francillon-Vieillot et al., 1990; Starck & Chinsamy, 2002; de Margerie, Cubo & Castanet, 2002; de Margerie et al., 2004; Chinsamy-Turan, 2005). In most of the long bones, fibrolamellar bone deposition continues right up to the periphery of the cortex indicating continuous fast growth at the time of death (Botha & Chinsamy, 2004; de Margerie et al., 2004; Bhat, Chinsamy & Parkington, 2019). Except for the ribs, growth marks or lines of arrested growth (Francillon-Vieillot et al., 1990; Castanet et al., 1993; Chinsamy et al., 1995) are absent within the fibrolamellar bone tissue suggesting uninterrupted rapid osteogenesis and fast growth early in ontogeny (Amprino, 1947; Francillon-Vieillot et al., 1990; Starck & Chinsamy, 2002; de Margerie, Cubo & Castanet, 2002; de Margerie et al., 2004; Chinsamy-Turan, 2005, 2012). However, in a recent study on *Anteosaurus*, Bhat, Shelton & Chinsamy, in press (b) reported four LAGs in an *Anteosaurus* femur BP/1/5591a. Absence/presence of growth marks/LAGs indicate a variable response to prevailing environmental conditions (Köhler et al., 2012; Chinsamy & Warburton, 2021) and give an indication of the ontogenetic status of the element (Chinsamy-Turan, 2005). The presence of multiple growth lines/marks indicates cyclical bone depositional rates during ontogeny (*e.g*., Amprino, 1947; de Margerie, Cubo & Castanet, 2002; de Margerie et al., 2004; Starck & Chinsamy, 2002). The proximal section of the rib BP/1/5591c has several rest lines in the outer periphery. These rest lines were also noticed in the femur SAM-PK-K291 of *Anteosaurus magnificus* by Bhat, Shelton & Chinsamy, in press (b). Presence of these peripheral rest lines indicate that the growth has significantly slowed down or periodically stopped at the time of death of the animal (Francillon-Vieillot et al., 1990; Castanet et al., 1993; Chinsamy et al., 1995; Starck & Chinsamy, 2002; Chinsamy-Turan, 2005; Erickson, 2005). These rest lines are related to the attainment of sexual and/or skeletal maturity (Horner, de Ricqlès & Padian, 1999, 2000; Chinsamy-Turan, 2005; Erickson, 2005; Cerda et al., 2017).

The cortex of *Anteosaurus magnificus* is composed of highly vascularized, uninterrupted fibrolamellar bone tissue surrounding the inner spongy medullary region, suggesting rapid growth early in ontogeny. However, the histology of two ribs and a previously described femur of another *Anteosaurus* taxon revealed interrupted growth pattern with the lines of arrested growth and peripheral rest lines, suggesting periodic interruptions in growth in this taxon, which may be correlated with an overall slowing down of growth during later stages of ontogeny. The absence of growth marks in the fibula of the same *Anteosaurus* sp. individual suggests intra-skeletal variability in growth. Peripheral rest lines were also noticed in the femur SAM-PK-K291 of *Anteosaurus magnificus* (Bhat, Shelton & Chinsamy, in press (b)). Thus, considering bone microstructure of the various skeletal elements studied from two *Anteosaurus* taxa, three stages of growth are noted for the genus *Anteosaurus*. The earliest stage of growth shows the deposition of highly vascularized fibrolamellar bone with woven matrix and numerous primary osteons. The presence of uninterrupted fibrolamellar bone tissue right up to the peripheral cortex in most of our specimens indicate a young ontogenetic status for the individuals from which the skeletal elements were sampled (*e.g.*, Ray, Chinsamy & Bandyopadhyay, 2005). During this stage, *Anteosaurus* had rapid growth without any interruption; however, textural shifts in the vascularization pattern occurred. Bone growth marks and secondary osteons are absent at this stage, further supporting their immature or sub-adult status (Ray, Chinsamy & Bandyopadhyay, 2005; Chinsamy-Turan, 2005). The second phase of growth was interrupted, which resulted in the deposition of LAGs as seen in *Anteosaurus* femur BP/1/5591a (Bhat, Shelton & Chinsamy, in press (b)). In this femur there is no associated change in degree or pattern of vascularization prior to or after the LAGs, *i.e*., LAGs were followed by resumption of bone deposition in the form of wide zones of rapidly formed fibrolamellar bone (Chinsamy & Rubidge, 1993; Horner, de Ricqlès & Padian, 2000). Growth slowed down significantly in the third phase as indicated by the deposition of numerous rest lines in the peripheral cortex which are accompanied by lamellar bone. Such changes in the histology are evident in rib BP/1/5591c as well as in femur SAM-PK-K291 of *Anteosaurus magnificus* (Kammerer, 2011) where the peripheral bone has just a few isolated primary osteons (Bhat, Shelton & Chinsamy, in press (b)). The differences in the histology of the different skeletal elements from single individuals suggests that *Anteosaurus* exhibited variability in how different parts of its skeleton grew.

Fibrolamellar bone tissue has been described in a wide variety of extinct and extant vertebrates (*e.g*., Enlow & Brown, 1957; Enlow, 1969; de Ricqlès, 1983; Chinsamy, 1990, 1995; Curry, 1999; Horner, de Ricqlès & Padian, 1999, 2000; de Ricqlès et al., 2000; Chinsamy-Turan, 2005; Chinsamy & Abdala, 2008, Chinsamy, Codorniú & Chiappe, 2009; Cerda & Chinsamy, 2012; Prondvai & Stein, 2014; Cerda, Chinsamy & Pol, 2014; Cerda et al., 2017), and has also been reported in the long bones of pelycosaurian-grade synapsids (Enlow & Brown, 1956, 1957, 1958; Enlow, 1969; de Ricqlès, 1974; Huttenlocker, Rega & Sumida, 2010; Shelton, 2015; Shelton et al., 2013; Shelton & Sander, 2017; Agliano, Sander & Wintrich, 2021), as well as in the limb elements of various therapsids (*e.g*., de Ricqlès, 1969, 1972, 1976; Chinsamy & Rubidge, 1993; Botha & Chinsamy, 2000; Ray, Botha & Chinsamy, 2004; Ray, Chinsamy & Bandyopadhyay, 2005; Ray, Mukherjee & Bandyopadhyay, 2009; Chinsamy-Turan, 2012; Botha, 2020). These findings suggest that this tissue appeared early in synapsid evolution, well before the origin of mammals.

### Histological variability

Bone histology of the multiple long bones of two *Anteosaurus* species revealed substantial variation in terms of degree of vascularization, tissue organization, amount of cancellous bone, presence/absence of peripheral lamellar bone tissue and incidence of lines of arrested growth. Such histological variation could be related to variations in the rate of periosteal deposition of different bones, ontogenetic age, varying amounts of cortical drift and remodeling, as well as to local conditions of growth within a single skeletal element (Enlow, 1963; de Margerie, Cubo & Castanet, 2002; de Margerie et al., 2004; Chinsamy-Turan, 2005; Cerda & Chinsamy, 2012). Since bone histology is influenced by phylogenetic, ontogenetic, functional and/or biomechanical constraints, such histological variations are expected and therefore indicate variable growth rates of skeletal elements (de Ricqlès, 1972; Chinsamy, 1990, 1995; de Ricqlès et al., 1991; Reid, 1996; Curry, 1999; Starck & Chinsamy, 2002; Chinsamy-Turan, 2005, 2012). When several elements of the same individual were examined, we had the opportunity to observe intra-skeletal variation. In all the bones, the medullary regions were secondarily remodeled and comprised of cancellous bone tissue, whereas the thick outer cortex consists of essentially primary fibrolamellar bone and subperiosteally lamellar bone. The cortex of femur SAM-PK-12088a is composed of a middle plexiform layer and a band of circumferential fibrolamellar bone. The latter is restricted to the outer periphery. Such a textural shift in vascularization may be related to dietary change during ontogeny, linked to increased ontogenetic age of the animal (Chinsamy et al., 2012; Cerda et al., 2017; Krupandan, Chinsamy-Turan & Pol, 2018). Differences in the vascular pattern have also been reported to be related to regional variation in the growth rate (de Margerie, Cubo & Castanet, 2002; de Margerie et al., 2004; de Margerie et al., 2005; de Cerff, Krupandan & Chinsamy, 2020). Another notable feature is the varying degree of secondary remodeling as well as the extent and distribution of cancellous bone among the different skeletal elements as well as within a single cross-section. The radius of *Anteosaurus magnificus* has an open medullary cavity with the least amount of cancellous tissue whereas the fibula and ribs of another *Anteosaurus* taxon has highly remodeled medullary regions, and the cortex of the proximal rib is completely porous. On the other hand, the midshaft section of another rib of the same individual displayed highly vascularized fibrolamellar bone tissue. These differences in the rib histology are most likely related to the location of the thin sections. In contrast to sauropod dinosaurs which exhibit a better growth mark record at the proximal end of the rib (Stein & Sander, 2009; Waskow & Sander, 2014), in anteosaurs the growth mark record at the diaphyseal and proximal regions remains consistent.

### Lifestyle adaptations

In addition to relative bone wall thickness and bone density, osteohistological characteristics have been used to deduce lifestyle adaptation of a variety of tetrapods (*e.g*. Wall, 1983; Chinsamy, 1997; de Ricqlès & de Buffrénil, 2001; Germain & Laurin, 2005; Gray et al., 2007; Houssaye, 2009; Canoville & Laurin, 2009, 2010; Laurin, Canoville & Germain, 2011; Hayashi et al., 2013; Nakajima, Hirayama & Endo, 2014; Houssaye et al., 2016; Canoville & Chinsamy, 2017; Montoya-Sanhueza & Chinsamy, 2018; Botha, 2020). Associated with semi-aquatic and aquatic lifestyles there is an increase in bone density, which counterbalances buoyancy (*e.g*. Chinsamy, 1991; Gray et al., 2007; Hayashi et al., 2013) and this is often reflected as a greater bone wall thickness (Wall, 1983). Interestingly, burrowing/fossorial animals also have thick bone walls (Bramble, 1982; Ultsch & Anderson, 1988; Lips, 1991; Magwene, 1993; Ray & Chinsamy, 2004; Botha & Chinsamy, 2004, 2005; Chinsamy & Abdala, 2008; Lyson et al., 2016; Montoya-Sanhueza & Chinsamy, 2017; Legendre & Botha-Brink, 2018; Bhat, Chinsamy & Parkington, 2019) as compared to terrestrial animals, which have lower relative bone thickness (RBT) values (<30%; *sensu* Wall, 1983), but given their large bulk, it is highly unlikely that anteosaurs were fossorial.

Among the anteosaur specimens studied here, except for the radius and the previously described femur (Bhat, Shelton & Chinsamy, in press (b)), most of the skeletal elements are characterized by thick bone walls and an extensive development of medullary spongiosa. However, femur SAM-PK-12088a does not have an extensively developed spongy medullary region, and the same applies to the radius (SAM-PK-12088b) of the same species which has a rather open medullary cavity with bone trabeculae. Similar bone tissues present in SAM-PK-12088a have been reported in the femur BP/1/5591a of the *Anteosaurus* by Bhat, Shelton & Chinsamy, in press (b).

The dicynodont *Lystrosaurus* has a very similar bone histology to the anteosaurs described here, and on the basis of its histology, Ray, Chinsamy & Bandyopadhyay (2005) proposed that it had a semi-aquatic/aquatic lifestyle. More recently using the same histological features, Botha (2020) proposed a fully terrestrial mode of life for *Lystrosaurus*. Botha (2020) further suggested biomechanical constraints to support the large body weight as the cause of the extensive development of medullary spongiosa. However, in a study of graviportal and aquatic tetrapods Houssaye et al. (2016) reported that the stylopodial bones and ribs increase bone compactness by reducing medullary cavity space. In particular, ribs showed more pronounced changes in compactness associated with aquatic behaviour than with graviportality, which supports the hypothesis that this microanatomical organization has a role in buoyancy control in shallow waters (Chinsamy, 1991; Gray et al., 2007; Hayashi et al., 2013; Houssaye et al., 2016), contra Botha (2020). Furthermore, contra Botha (2020), elephants (Houssaye et al., 2016), giraffes (Smith, 2020), and bison (Sander & Andrassy, 2006; Houssaye et al., 2016; Canoville, de Buffrénil & Laurin, 2016), which are large, graviportal terrestrial animals do not have medullary cavities infilled with spongious bone tissue as a biomechanical adaption for their bulk. However, as in *Anteosaurus*, the ribs of the known aquatic reptile, *Claudiosaurus* (de Buffrénil & Mazin, 1989) show a complete infilling of the medullary region by cancellous bone and are considered pachyostotic as an adaptation for their semi-aquatic lifestyles (de Ricqlès & de Buffrénil, 2001).

It is also worth mentioning that Middle Permian pareiasaurs, which were equally large extinct tetrapods contemporaneous with dinocephalians, exhibited medullary spongiosa and thin compact cortices (Canoville & Chinsamy, 2017). Isotopic analysis of the teeth of dinocephalians and pareiasaurs has shown that these large graviportal animals inhabited different ecological niches during Middle and Late Permian times with pareiasaurs sharing a terrestrial habitat with therocephalians. (Canoville, Thomas & Chinsamy, 2014), although Rey et al. (2020) subsequently reported low oxygen values for anteosaurs which point towards water dependency. However, they further suggested that a larger sample size is needed to verify their lower isotopic signatures (Rey et al., 2020) and supported a previously proposed terrestrial lifestyle for anteosaurs. Thus, based on the osteological features and oxygen isotopic signatures of teeth and bones, *Anteosaurus* is regarded as a fully terrestrial animal (Kammerer, 2011; Sennikov, 1996; Van Valkenburgh & Jenkins, 2002; Rey et al., 2020). However, the mixed histological features present in the skeletal elements and especially the ribs point towards aquatic tendencies. We cautiously propose that it is likely that *Anteosaurus* may have occasionally inhabited ephemeral water ponds like modern semi-aquatic *Hippopotamus* (Houssaye et al., 2016), but we suggest that in future more comprehensive sampling should be undertaken to verify our conclusions.

## Conclusions

Three stages of growth dynamics are deduced for the genus *Anteosaurus*: the first stage during early ontogeny is rapid, the second stage has periodic interruptions (LAGs) in the bone deposition, while the third stage shows a marked slowing down of bone deposition and overall growth as indicated by the presence of several peripheral rest lines. Most of the skeletal elements are characterized by thick bone walls, extensive secondary reconstruction and the complete infilling of the medullary cavity. The radius of *Anteosaurus magnificus* and previously studied femur of *Anteosaurus* sp. have open medullary cavities with struts of bony trabeculae. Based on the mixed histological features evident in the skeletal elements studied (*i.e*., complete infilling of medullary cavity in some elements and open medullae in others), and considering the previous oxygen isotopic studies (Canoville, Thomas & Chinsamy, 2014), we propose that *Anteosaurus* may have been primarily terrestrial but it may have occasionally occupied ephemeral ponds as compared to the contemporaneous fully terrestrial pareiasaurs.

## Supplemental Information

10.7717/peerj.12082/supp-1 Supplemental Information 1 Skeletal elements of Anteosaurus specimens studied. All the material was recovered from the Tapinocephalus Assemblage Zone (Beaufort Group, Karoo Supergroup), South Africa. Abbreviations: SAM, Iziko South African Museums, Cape Town, South Africa; BP, Evol

## Additional Information and Declarations

### Field Study Permissions

The following information was supplied relating to field study approvals (*i.e*., approving body and any reference numbers): Permission to section the fossils was obtained from the South African Heritage Resources Agency (SAHRA; Permits 2076, 2131, and 3752-4658).

### Data Availability

The following information was supplied regarding data availability: All the specimens and the resulting thin section slides are housed in the Iziko Museums of Cape Town, and the Evolutionary Studies Institute (formerly the Bernard Price Institute) at the University of the Witwatersrand, Johannesburg, South Africa: SAM-PK-12088a, SAM-PK-12088b, SAM-PK-12088c, SAM-PK-12088d, BP/1/5591b, BP/1/5591c, BP/1/5591d.
